# Association between lipid-lowering agents and severe hyponatremia: a population-based case–control study

**DOI:** 10.1007/s00228-020-03006-8

**Published:** 2020-11-19

**Authors:** Jakob Skov, Henrik Falhammar, Jan Calissendorff, Jonatan D Lindh, Buster Mannheimer

**Affiliations:** 1 Department of Molecular Medicine and Surgery, Karolinska Institutet, Stockholm, Sweden; 2 Department of Medicine, Karlstad Central Hospital, Karlstad, Sweden; 3 Department of Endocrinology, Metabolism and Diabetes, Karolinska University Hospital, Stockholm, Sweden; 4 Department of Laboratory Medicine, Division of Clinical Pharmacology, Karolinska University Hospital Huddinge, Karolinska Institutet, Stockholm, Sweden; 5 Department of Clinical Science and Education, Södersjukhuset, Karolinska Institutet, Stockholm, Sweden

**Keywords:** Hospitalization, Statin, Ezetimibe, SIADH, Adverse reaction, Electrolyte

## Abstract

**Purpose:**

Drug-induced hyponatremia is common, with medications from many drug-classes implicated. Lipid-lowering agents are among the most prescribed drugs. Limited evidence suggests an inverse association between statins and hyponatremia, while data on other lipid-lowering agents is absent. The objective of this investigation was to study the association between lipid-lowering drugs and hospitalization due to hyponatremia.

**Methods:**

This was a register-based case–control study of the general Swedish population. Those hospitalized with a main diagnosis of hyponatremia (*n* = 11,213) were compared with matched controls (*n* = 44,801). Multivariable logistic regression adjusting for co-medication, diseases, previous hospitalizations, and socioeconomic factors was used to explore the association between severe hyponatremia and the use of lipid-lowering drugs.

**Results:**

Unadjusted ORs (95% CI) for hospitalization due to hyponatremia were 1.28 (1.22–1.35) for statins, 1.09 (0.79–1.47) for ezetimibe, 1.38 (0.88–2.12) for fibrates, and 2.12 (1.31–3.35) for resins. After adjustment for confounding factors the adjusted odds ratios (95% CI) compared with controls were 0.69 (0.64–0.74) for statins, 0.60 (0.41–0.86) for ezetimibe, 0.87 (0.51–1.42) for fibrates, and 1.21 (0.69–2.06) for resins.

**Conclusions:**

Use of statins and ezetimibe was inversely correlated with severe hyponatremia. Consequently, these drugs are unlikely culprits in patients with hyponatremia, and they appear safe to initiate in hyponatremic patients. A potential protective effect warrants further studies on how statins and other lipid-lowering drugs are linked to dysnatremias.

**Electronic supplementary material:**

The online version of this article (10.1007/s00228-020-03006-8) contains supplementary material, which is available to authorized users.

## Introduction

Hyponatremia, usually defined as a serum sodium below 135 mmol/L, is the most common electrolyte disturbance, affecting between 10 and 30% of hospitalized patients [1]. Acute symptomatic hyponatremia is a rare condition presenting with symptoms such as severe nausea, seizures, coma, and ultimately death if prompt treatment is not initiated [2]. In chronic hyponatremia, the clinical panorama is more inconspicuous, but fatigue, gait instability, and cognitive deficits are common [3, 4]. Despite milder symptoms, chronic hyponatremia is a marker of poor clinical outcome, associated with longer durations of stay [5], higher readmission rates [6], and increased risk of death from underlying diseases [7, 8] when compared with normonatremic patients. For unknown reasons, the risk of death appears to be more pronounced in men than in women [9]. The most common cause of hyponatremia is medications [10], with drugs across most clinical fields implicated in hyponatremia [11–15]. A select few drugs, primarily antidiabetic agents [16] and lithium [17] have also been associated with a reduced risk of hyponatremia, whereas evidence is missing or inconclusive for most compounds.

In theory, statins could cause fluid retention and hyponatremia through a mechanism similar to syndrome of inappropriate antidiuretic hormone secretion (SIADH), as they have been shown to increase renal expression of aquaporin-2 independent of arginine vasopressin and to reduce diuresis in rat models with central diabetes insipidus [18]. Observational data on patients with lithium-induced diabetes insipidus, indicating different urine osmolality in statin users and non-users, has also been published [19]. However, despite their frequent use, data on lipid-lowering agents and hyponatremia is scarce. Searching VigiBase, the World Health Organization global database of individual case safety reports [20] from inception up to July 10, 2020, returns 374 reports of hyponatremia as a suspected adverse reaction of treatment with statins (simvastatin, atorvastatin, rosuvastatin, pravastatin, fluvastatin, lovastatin) or ezetimibe. This corresponds to 0.1% of submitted reports for these compounds. To the best of our knowledge, no studies reporting on increased risk of hyponatremia in patients treated with statins have been published. To the contrary, the limited data available suggests a modest inverse association between statins and hyponatremia [21–23]. while data on other lipid-lowering agents is absent. This could signal that these drugs are safe, or even offer limited protection from hyponatremia, but current data is insufficient to support such conclusions.

Hence, the aim of this study was to explore the association between treatment with lipid-lowering agents and hospitalization due to hyponatremia.

## Methods

This was a retrospective population–based case–control study on the adult Swedish population. Cases were defined as subjects, 18 years or older, in the National Patient Register (NPR) [24], hospitalized with a first-ever main diagnosis of hyponatremia (E87.1) or SIADH (E22.2) between October 1, 2005, and December 31, 2014. A first ever diagnosis was defined as absence of a prior diagnosis (main or secondary) of E87.1 or E22.2 dating back to January 1, 1997. For every case, four controls without a previous diagnosis of hyponatremia (main or secondary diagnosis since January 1, 1997) were randomly identified from the Total Population Register[24]. Controls were matched for age, sex, and municipality. During this time-period (January 1, 1997, to December 31, 2014), all diagnoses in the NPR were recorded according to the International Classification of Diseases, Tenth Revision (ICD-10). Concurrent and previous use of medications was identified using the Swedish Prescribed Drug Register (SPDR), which contains individual data on all prescription drugs dispensed in Sweden, dating back to July 1, 2005 [25]. The longitudinal integration database for health insurance and labour market studies register (LISA) was used to collect data on socioeconomic status [26]. The recruitment process has been described in more detail in a previous work [27]. The study was approved by the Regional Ethical Review Board in Stockholm. Informed consent was waived.

### Variables

Variables of interest (lipid-lowering agents) identified by corresponding Anatomical Therapeutic Chemical (ATC) codes along with other exposure variables are listed in Table 1. Drug exposure was defined as a documented dispensing within 90 days prior to the index date, i.e., the date of hospitalization due to hyponatremia. Confounding factors accounted for in the statistical analysis included concurrent medications, socioeconomic factors, and medical conditions identified by collating information from the NPR, the SPDR, and LISA. Lipid-lowering therapy is often initiated shortly after cardiovascular events (secondary prevention). In an effort to optimize adjustment of confounders we therefore treated cardiovascular events occurring within 90 days of a main diagnosis of hyponatremia, versus older events, as separate confounders. Due to the large number of variables, intermediate models gradually adjusting for confounding factors were calculated by adding variables for comorbid conditions, for concurrent medications, and for socioeconomic factors and frailty to the crude model in a step-by step fashion. Finally, in a post hoc analysis exploring the combined effect of statins and ezetimibe, we considered use of statins only, ezetimibe only, and the combined use of statins and ezetimibe as three independent (non-interacting) variables.

**Table 1:** Variables included in the multiple logistic regression analysis and their definition

Variables	Codes
Drugs of primary interest	ATC codes beginning with:
Lipid-lowering agents	C10
Statins	C10AA, C10B
Simvastatin	C10AA01, C10BA02, C10BA04
Lovastatin	C10AA02, C10BA01
Pravastatin	C10AA03, C10BA03
Fluvastatin	C10AA04,
Atorvastatin	C10AA05, C10BA05
Rosuvastatin	C10AA07, C10BA06
Fibrates	C10AB, C10BA03, C10BA04
Bezafibrate	C10AB02
Gemfibrozil	C10AB04
Fenofibrate	C10AB05, C10BA03, C10BA04
Resins	C10AC
Cholestyramine	C10AC01
Colestipol	C10AC02
Colesevelam	C10AC04
Nicotinic acid	C10AD
Nicotinic acid	C10AD02, C10AD52
Cholesterol absorption inhibitor	C10AX09, C10BA02, C10BA05, C10BA06
Ezetimibe	C10AX09, C10BA02, C10BA05, C10BA06
Concurrent medications	
Antiepileptic drugs	
Carbamazepine	N03AF01
Oxcarbazepine	N03AF02
Phenytoin	N03AB02
Valproate	N03AG01
Lamotrigine	N03AX09
Levetiracetam	N03AX14
Gabapentin	N03AX12
Diuretics and drugs acting on the renin–angiotensin system	
Furosemide	C03C
Thiazides	C03A, C09BA, C09DA, C03EA
Agents acting on the renin–angiotensin system	C09
Antibiotics	
Fluoroquinolones	J01MA
Macrolides	J01FA
Trimethoprim sulfamethoxazole	J01EE
Antidepressants	
Serotonin reuptake inhibitors	N06AB
Tricyclic antidepressants	N06AA
Other antidepressants	N06AX
Other drugs	
Amiodarone	C01BD01
Desmopression	H01BA02
Antipsychotics	N05A excluding N05AN
Lithium	N05AN
Proton pump inhibitors	A02BC, A02BD06
Comorbidities	ICD10 codes beginning with:
Renal diseases	
Renal insufficiency	N17-19, procedure codes DR016, DR024, KAS00, KAS10, KAS20
Infections	
Sepsis	A41 ≤90 days from index date
Pneumonia	J18 ≤ 90 days from index date
Meningitis	G00–G07 ≤ 90 days from index date
Heart and vascular diseases	
Ischaemic heart disease, recent	I20–24 ≤ 90 days from index date
Ischaemic heart disease, old	I20–24 > 90 days from index date, I25
Congestive heart failure	I50
Cerebrovascular diseases, recent	I60–64 ≤ 90 days from index date
Cerebrovascular diseases, old	I60–64 > 90 days from index date, I69
Gastrointestinal diseases	
Pancreatic disease	K85, K860-1
Inflammatory bowel disease	K50–51
Liver diseases	K70–77 procedure codes JJB, JJC
Other diseases	
Hypothyroidism	E03, E06.3
Malnutrition	E43.9, E41.9
COPD	J44
Pulmonary embolism	I26
Malignancy	C
	Combination of ATC and ICD10 codes, each beginning with:
Alcoholism	ATC: N07BB03, N07BB04, N07BB01, N07BB05, N07BB ICD10: E244, F10, G312, G621, G721, I426, K292, K70, K860, O354, P043, Q860, T51, Y90–91, Z502, Z714
Adrenal insufficiency	ATC: H02AA, H01BA ICD10: E27.1, E27.2, E27.3, E27.4, E25
Diabetes mellitus	ATC: A10 ICD10: E10–E14
Socioeconomic factors/frailty	
Education	Increasing levels of education from 1 to 6, ordinal variable
Income	Annual income in Swedish krona, continuous variable
Unemployment	Number of days, continuous variable
Drug use	Number of dispensed drugs 90 days prior to index date, categorized into < 4, 4–7, 8–12, and > 12 drugs
Duration of hospitalization	≥ 3 days

*ATC* Anatomical Therapeutic Chemical, *COPD* chronic obstructive pulmonary disease, *ICD* International Classification of Diseases

### Statistical analysis

To study the association between hyponatremia and lipid-lowering agents, we used univariable and multivariable logistic regression. The association between hyponatremia and the different lipid-lowering agents was presented as crude and adjusted (for potential confounders) odds ratios (OR) with 95 confidence intervals. A *p* < 0.05 was considered significant. All calculations were performed using R version 3.6.1 [28].

## Results

**Fig. 1:**
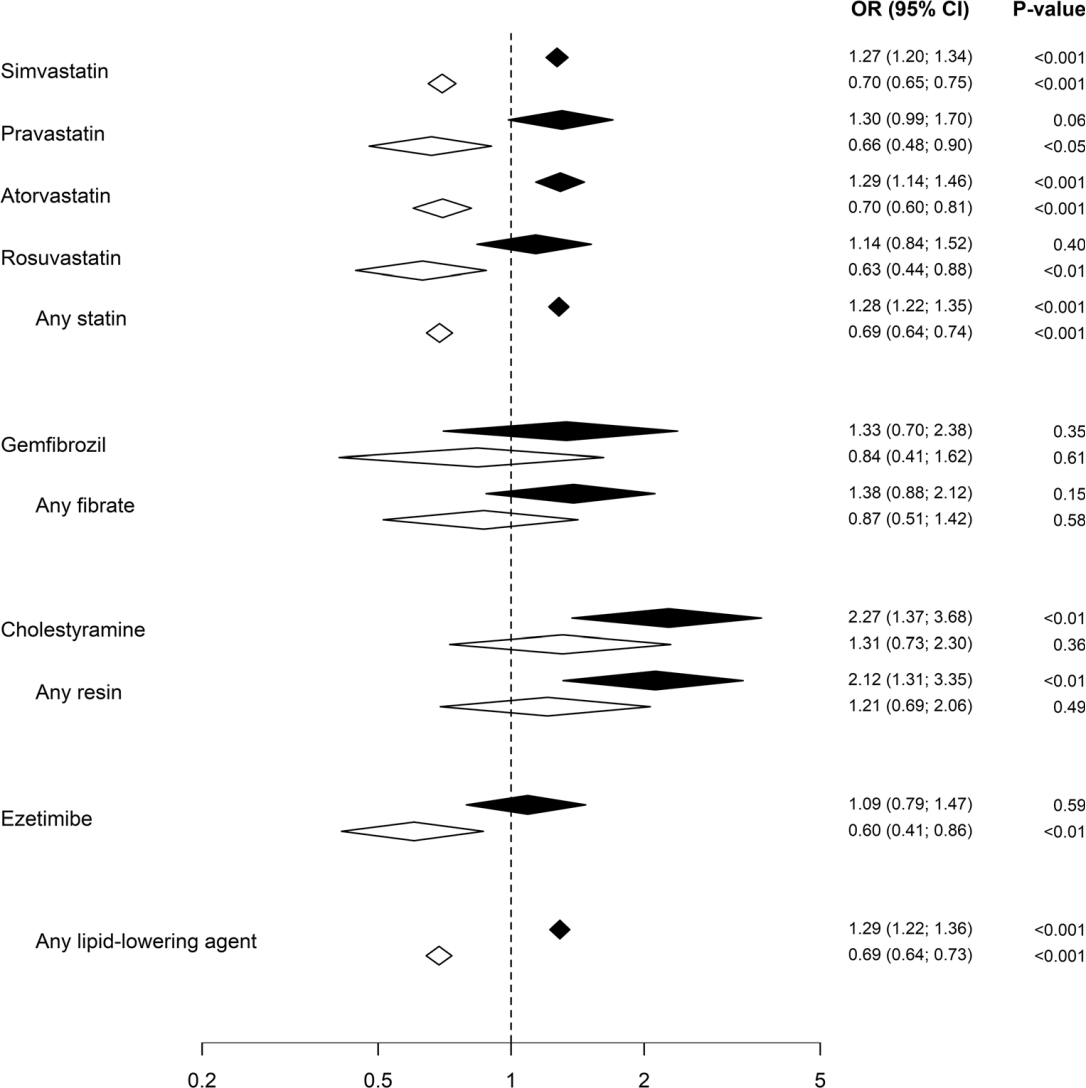
Unadjusted (black diamonds) and adjusted (white diamonds) odds ratios (ORs), including 95% confidence intervals (95% CI) for hospitalization due to hyponatremia in patients using lipid-lowering agents. The following substances had few users that are therefore not presented separately but are included in calculations at drug-class level: fluvastatin and lovastatin (statins), bezafibrate and fenofibrate (fibrates), colesevelam, and colestipol (resins). Nicotinic acid, used by two subjects, is included in calculations on the overall effect of any lipid-lowering agent

In total, 35,469 individuals were hospitalized with a first-ever diagnosis of hyponatremia during the study period. Of those, 24,191 individuals with hyponatremia as a secondary diagnosis and 65 individuals under the age of 18 at the index date were excluded, yielding a final sample of 11,213 individuals with a main diagnosis of hyponatremia. For every case, four controls (*n* = 44,801) were matched at the index date. The median age in the total cohort was 76 years (interquartile range 65–84 years), with women accounting for 72%. Clinical characteristics of cases and matched controls, alongside with data on use of lipid-lowering agents, are presented in Table 2. Overall, cases carried a greater comorbid burden than their matched controls. They used more medications and were more likely to have a history of inpatient care. The most common concomitant diseases included hypertension, ischemic heart disease, diabetes, alcoholism, congestive heart failure, cerebrovascular disease, and chronic obstructive pulmonary disease. Statins were by far the most commonly used lipid-lowering agents, with 20.1% of cases (*n* = 2,249) and 16.4% of controls (*n* = 7,333) on statin therapy. The second most common drug was ezetimibe. Resin-prescriptions were largely accounted for by cholestyramine, usually prescribed as a bile acid sequestrant rather than a lipid-lowering agent in Sweden. Unadjusted ORs for hospitalization due to hyponatremia ranged from 1.09 (95% CI 0.79–1.47) for ezetimibe to 2.12 (95% CI 1.31–3.35) for resins. After adjustment for confounding factors, ORs decreased considerably, ranging from 0.60 (95% CI 0.41–0.86) for ezetimibe, 0.69 (95% CI 0.64-0.74) for statins, 0.87 (95% CI 0.51–1.42) for fibrates to 1.21 (95% CI 0.69–2.06) for resins. The unadjusted and adjusted associations are presented in Fig. 1.

**Table 2:** Medical characteristics (selection of variables from Table 1) and use of lipid-lowering agents

	Number (%) of total cases (n=11,213)	Number (%) of total controls (n=44,801)
Diagnosis		
Malignancy	3096 (27.6)	9149 (20.4)
Ischemic heart disease, recent^1^	498 (4.4)	405 (0.9)
Ischemic heart disease, old^2^	1918 (17.1)	6072 (13.6)
Diabetes mellitus	1939 (17.3)	5277 (11.7)
Alcoholism	1764 (15.7)	833 (1.9)
Congestive heart failure	1453 (13.0)	3533 (7.9)
Cerebrovascular disease, recent	218 (1.9)	164 (0.3)
Cerebrovascular disease, old	1299 (11.6)	3410 (7.6)
COPD	1125 (10.0)	1576 (3.5)
Hypothyroidism	1139 (10.2)	1994 (4.5)
Renal disease	489 (4.4)	888 (2.0)
Adrenal insufficiency	460 (4.1)	300 (0.7)
Liver disease	421 (3.8)	332 (0.7)
Pancreatic disease	252 (2.2)	395 (0.9)
IBD^4^	221 (2.0)	444 (0.1)
Medications		
Antidepressants	2817 (25.1)	5745 (12.8)
Antipsychotics	772 (6.9)	1096 (2.4)
Antiepileptic drugs	1061 (9.5)	1128 (2.5)
Furosemide	1735 (15.5)	5487 (12.2)
Thiazide diuretics	4364 (38.9)	6103 (13.6)
Proxy for frailty		
Number of dispensed drugs 90 days prior to index date		
<4 drugs	2215 (19.8)	22,892 (51.1)
4-7 drugs	3421 (30.5)	12,967 (28.9)
8-12 drugs	3558 (31.7)	7010 (15.6)
>12 drugs	2019 (18.0)	1.932 (4.3)
Number of patients with ≥1 hospitalization ≥3 days duration	4852 (43.2)	9477 (21.2)
Lipid-lowering medications		
Any lipid-lowering drug	2314 (20.6)	7525 (16.7)
Statins	2249 (20.1)	7333 (16.4)
Simvastatin	1823 (16.2)	5940 (13.3)
Pravastatin	69 (0.6)	212 (0.5)
Fluvastatin	1 (<0.1)	11 (<0.1)
Atorvastatin	324 (2.9)	1009 (2.3)
Rosuvastatin	56 (0.5)	197 (0.4)
Lovastatin	0 (0)	0 (0)
Fibrates	27 (0.2)	78 (0.2)
Bezafibrate	8 (0.1)	23 (0.1)
Gemfibrozil	14 (0.1)	42 (0.1)
Fenofibrate	5 (<0.1)	13 (<0.1)
Resins	27 (0.2)	51 (0.1)
Cholestyramine	25 (0.2)	44 (0.1)
Colestipol	2 (<0.1)	7 (<0.1)
Colesevelam	0 (0.0)	0 (0.0)
Nicotinic acid	0 (0.0)	2 (<0.1)
Ezetimibe	51 (0.4)	187 (0.4)

^1^Within 90 days of index date; ^2^more than 90 days from index date; ^3^chronic obstructive pulmonary disease; ^4^inflammatory bowel disease

Results of the intermediate models demonstrated a gradual reduction in crude odds ratios after first adjusting for comorbidities and then for concurrent medications, but only a marginal effect from adjusting for socioeconomic factors (Supplementary materials Table S1). In the post-hoc analysis, after adjustment for confounders, ORs were 0.69 (95% CI 0.64–0.74) for statins, 0.54 (95% CI 0.31–0.90) for ezetimibe, and 0.45 (95% CI 0.27–0.73) for use of both statins and ezetimibe.

## Discussion

In this case–control study on the general Swedish population, we demonstrated that use of statins and ezetimibe was inversely correlated with hospitalization due to hyponatremia after adjusting for multiple confounders. For resins and fibrates results were inconclusive.

Lipid-lowering agents are among the most prescribed drugs today, with more than 40% of elderly (> 65 years) US citizens using statins [29]. This is also the age-group most often affected by hyponatremia, with serum sodium levels below 135 mmol/L found in up to 20% of elderly during hospitalization [30]. Despite their co-occurrence, surprisingly little is known about statins and other lipid-lowering agents in relation to hyponatremia. Instead, the absence of evidence [11] is sometimes used as evidence of absence in clinical practice, despite the inherent risk with such strategies. Here, we report an inverse association between severe hyponatremia and the use of statins and ezetimibe. For fibrates, results were similar, but did not reach statistical significance. By contrast, resins showed a non-significant increase in risk of hyponatremia, but for resins, results are difficult to interpret in this context, as cholestyramine, a drug primarily used to treat gastrointestinal diseases, accounted for most prescriptions.

Our findings on statins are corroborated by a recent study by Israel et al., reporting an inverse association between statin-use and hyponatremia in participants from the SPRINT study [21]. The potential mechanism by which statins and ezetimibe could potentially reduce the risk of severe hyponatremia is unknown. The similarity in magnitude and direction of association observed here could imply that the correlation is explained by alterations in lipid levels rather than by the drugs themselves. However, this explanation is contradicted by the study by Israel et al. showing that statin use was inversely associated with the development of hyponatremia after adjustment for multiple factors, including C-HDL levels [21]. Statins are known to exert pleiotropic effects beyond inhibition of 3-hydroxy-3-methylglutaryl coenzyme A [31], and short-term exposure to atorvastatin has been shown to decrease fractional urinary excretion of sodium in healthy subjects and in individuals with type-2 diabetes. This may therefore constitute a mechanism [32, 33]. Nevertheless, the most reasonable explanation to a potential inverse association is perhaps that the preservation of glomerular filtration rate associated with use of lipid-lowering agents [34, 35] also slows the decline in capacity for electrolyte free water clearance, thus reducing the risk of fluid retention and hyponatremia. In the post hoc analysis, the OR for combined use of both statins and ezetimibe was lower than the OR for use of either compound alone, consistent with an additive effect. This observation does not provide obvious clues to the underlying mechanism, as it is consistent with both direct effects from the drugs, and indirect effects from altered lipid-profiles, but it does support an inverse association between statins and hyponatremia, as do findings from smaller studies similar to ours [21–23]. Still, pending additional data in support of a protective effect from lipid-lowering drugs with regard to hyponatremia, the most prudent conclusion is perhaps that lipid lowering agents, statins and ezetimibe in particular, are unlikely to cause or contribute to severe hyponatremia.

### Limitations

The main limitation of this study is the lack of laboratory data, plasma sodium concentrations in particular, that are not recorded in the Swedish national health registries. It is important to note that the sensitivity for hyponatremia (serum sodium < 135 mmol/L) using diagnostic records is poor [36]. On the other hand, with the clinical panorama of hyponatremia ranging from subtle to severe symptoms, using a main diagnosis of hyponatremia rather than secondary diagnoses, diagnoses made in outpatient care, or laboratory data to define cases, allowed us to limit cases to patients with clinically relevant hyponatremia, and ascertain that hyponatremia was indeed the condition that first and foremost motivated inpatient care. This was demonstrated in a previous validation showing that 89% of subjects receiving a main diagnosis of hyponatremia had been hospitalized primarily due to symptoms of hyponatremia and that the mean plasma sodium concentration (adjusted for glucose) was 121 mmol/L [27]. This, in combination with a population-based design including all Swedish residents during almost a decade, is a major strength compared with smaller studies. The finding of a reversed association may seem surprising and raise the question of potential bias attributed to the methodology. The cohort used in the present study has indeed previously been used to further characterize several groups of drugs known to increase the risk for hyponatremia using a similar design. Thus, newly initiated serotonergic antidepressants were associated with markedly elevated adjusted ORs of around 5 [27]. The increased risk attributed to antiepileptic was even larger [13]. However, the fact that our methodology is capable of addressing increased associations does not exclude the possibility of bias distorting the results. Many different disease mechanisms, sometimes overlapping, can lead to hyponatremia. Our model therefore included a wide range of concurrent illnesses and medications with known association with hyponatremia. In addition, we also included socioeconomic factors and frailty, variables that are linked to several types of severe disease. For example, socioeconomic status is linked to alcoholism and smoking, variables that are not sufficiently reflected in the registers used, which in turn may contribute to residual confounding. However, according to the stepwise model, socioeconomic factors only had a marginal effect on hospitalization (Supplementary materials Table S1). Matching hospitalized patients with population controls introduces the risk of information bias. This risk is most apparent with the NPR, as this register includes diagnoses collected from hospital associated care only (inpatient and outpatient), whereas the SPDR, which includes information on drugs prescribed in primary health care as well, is less sensitive to this bias. The inclusion of numerous variables could potentially introduce bias due to over-adjustment. However, the one-in-ten-rule suggests that one variable could be included for every ten cases included without running the risk of overfitting. Over 11,000 cases in the current study may therefore motivate the use of 61 independent variables and still keeping risk of over fitting low. For increased understanding of what factors were responsible for the considerable shift from significantly increased to significantly decreased risk of hyponatremia we included intermediate models that built on the crude model by step-by-step adding of confounders in groups of comorbidities, concurrent medications, and socioeconomic factors. As evident from the results, adjusting for medications appeared most important (supplemental Table 1). Furthermore, defining drug-use by prescriptions filled within the last 90 days may have resulted in some misclassifications, as statin-users are known to show large variation in persistence with many people stopping and starting treatment [37]. Finally, the study spans over a long period of time. An additional source of potential residual confounding may come from changes in prescribing patterns for example due to the new reimbursement scheme for lipid lowering agents introduced in 2009 [38]. The effects of such time-dependent effect are however difficult to predict.

In conclusion, we found an inverse association between statins/ezetimibe and hospitalization due to hyponatremia. The implication of these observations is that most lipid-lowering agents are likely to be safe for continued use in patients with hyponatremia of unknown origin, and safe to initiate, even in patients at high risk of hyponatremia. Causal relationships cannot be established with certainty using observational data, but a potential protective effect warrants further studies on how statins and other lipid-lowering drugs are linked to dysnatremias.

## Electronic supplementary materials

Table S1 A complete list of variables included in the multivariate logistic regression model (DOCX 29.3 kb).

ESM 1 (PDF 48 kb).

## Data Availability

Data will be made available upon reasonable requests.
