# Supplementary material for: Association between lipid-lowering agents and severe hyponatremia: a population-based case–control study
Source: Eur J Clin Pharmacol. 2020 Nov 19;77(5):747–55. doi: 10.1007/s00228-020-03006-8 (PMC8032630; doi:10.1007/s00228-020-03006-8)
Supplement: Supplementary file 1 — A complete list of variables included in the multivariate logistic regression model (DOCX 29.3 kb). [file 228_2020_3006_MOESM1_ESM.docx]

**Table S1.**

|  | Model 1  OR (95% CI) | Model 2  OR (95% CI) | Model 3  OR (95% CI) | Model 4  OR (95% CI) |
| --- | --- | --- | --- | --- |
| Any lipid-lowering drug | 1.29 (1.22-1.36) | 1.14 (1.07-1.21) | 0.69 (0.64-0.73) | 0.69 (0.64-0.73) |
| Statins | 1.28 (1.22-1.35) | 1.14 (1.07-1.21) | 0.69 (0.64-0.74) | 0.69 (0.64-0.74) |
| Simvastatin | 1.27 (1.20-1.34) | 1.14 (1.07-1.22) | 0.70 (0.65-0.75) | 0.70 (0.65-0.75) |
| Pravastatin | 1.30 (0.99-1.70) | 1.26 (0.93-1.67) | 0.66 (0.48-0.90) | 0.66 (0.48-0.90) |
| Atorvastatin | 1.29 (1.14-1.46) | 1.14 (0.99-1.31) | 0.70 (0.60-0.81) | 0.70 (0.60-0.81) |
| Rosuvastatin | 1.14 (0.84-1.52) | 1.06 (0.77-1.46) | 0.64 (0.45-0.88) | 0.63 (0.44-0.88) |
| Fibrates | 1.38 (0.88-2.12) | 1.25 (0.77-1.99) | 0.89 (0.53-1.45) | 0.87 (0.51-1.42) |
| Gemfibrozil | 1.33 (0.70-2.38) | 1.23 (0.62-2.29) | 0.86 (0.44-1.68) | 0.84 (0.41-1.62) |
| Resins | 2.12 (1.31-3.35) | 1.36 (0.80-2.27) | 1.21 (0.69-2.06) | 1.21 (0.69-2.06) |
| Cholestyramine | 2.27 (1.37-3.68) | 1.39 (0.79-2.38) | 1.31 (0.72-2.29) | 1.31 (0.73-2.30) |
| Ezetimibe | 1.09 (0.79-1.47) | 0.90 (0.64-1.25) | 0.59 (0.41-0.85) | 0.60 (0.41-0.86) |

Model 1: unadjusted odds ratios; Model 2: odds ratios adjusted for comorbidities; Model 3: odds ratios adjusted for comorbidities and concurrent medications; Model 4: odds ratios adjusted for comorbidities, concurrent medications and socioeconomic factors/frailty. Model 4 is synonymous with the adjusted odds ratios as presented in the article.
