# Supplementary material for: Association between lipid-lowering agents and severe hyponatremia: a population-based case–control study
Source: Eur J Clin Pharmacol. 2020 Nov 19;77(5):747–55. doi: 10.1007/s00228-020-03006-8 (PMC8032630; doi:10.1007/s00228-020-03006-8)
Supplement: Supplementary file 2 — (PDF 48 kb). [file 228_2020_3006_MOESM2_ESM.pdf]

Supplementary table provided demonstrate the changes in estimates due to the two errors. Nb that the provided table is only to be used for the review and not to be included in the manuscript nor tables to be published.

	CORRECTED Crude OR (95% CI)	INCORRECT Crude OR (95% CI)	CORRECTED Adj. OR (95% CI)	INCORRECT Adj. OR (95% CI)
Any lipid-lowering drug	1.29 (1.22-1.36)	1.27 (1.21-1.33)	0.69 (0.64-0.73)	0.64 (0.60-0.68)
Statins	1.28 (1.22-1.35)	1.26 (1.20-1.32)	0.69 (0.64-0.74)	0.64 (0.60-0.68)
Simvastatin	1.27 (1.20-1.34)	1.25 (1.19-1.32)	0.70 (0.65-0.75)	0.65 (0.61-0.70)
Pravastatin	1.30 (0.99-1.70)	1.27 (0.99-1.60)	0.66 (0.48-0.90)	0.63 (0.47-0.83)
Atorvastatin	1.29 (1.14-1.46)	1.29 (1.15-1.44)	0.70 (0.60-0.81)	0.64 (0.55-0.73)
Rosuvastatin	1.14 (0.84-1.52)	1.17 (0.89-1.53)	0.63 (0.44-0.88)	0.68 (0.50-0.93)
Fibrates	1.38 (0.88-2.12)	1.31 (0.86-1.92)	0.87 (0.51-1.42)	0.69 (0.42-1.08)
Gemfibrozil	1.33 (0.70-2.38)	1.49 (0.86-2.48)	0.84 (0.41-1.62)	0.81 (0.43-1.48)
Resins	2.12 (1.31-3.35)	2.31 (1.53-3.45)	1.21 (0.69-2.06)	1.28 (0.79-2.05)
Cholestyramine	2.27 (1.37-3.68)	2.45 (1.57-3.75)	1.31 (0.73-2.30)	1.33 (0.79-2.20)
Ezetimibe	1.09 (0.79-1.47)	1.10 (0.82-1.46)	0.60 (0.41-0.86)	0.55 (0.38-0.77)
